# Editorial: In celebration of women in science: Structural biology

**DOI:** 10.3389/fmolb.2023.1174561

**Published:** 2023-04-18

**Authors:** Roberta Spadaccini

**Affiliations:** Department of Science and Technology, Università Degli Studi Del Sannio, Benevento, Italy

**Keywords:** structural biology, tauopathies, ferritin, apoptosis, vitallogenin, BLH3 motif

Why do we still have to celebrate women in science, why are women in science not a normality? For decades women have been underrepresented in STEM (science, technology, engineering, and mathematics) at universities and occupations. As a matter of fact only around 30% of researchers globally are female. Despite this huge imbalance, women have given a huge contribute to STEM research in general and to the development of Structural Biology in particular. Women were among structural biology’s earliest pioneers. The work of Rosalind Franklin, a British crystallographer, laid the basis for the understanding of the double helix structure of DNA. Crystallographers are also two of the eight women scientists who won the Nobel prize for Chemistry, Dorothy Hodgkin awarded in 1964 for her pioneering work in X-ray crystallography of biomolecules and Ada Yonath who won it in 2009 for her studies on the structure and function of the ribosome. Structural studies of Cas9 were tremendously important for Jennifer Doudna and Emmanuelle Charpentier who were awarded with the 2020 Chemistry Nobel prize for their work on the gene editing tool CRISPR/Cas9.

The works presented in this Research Topic give a small account of the diversity of research performed by women across the entire breadth of Structural Biology research and present, advances in compelling problems like protein aggregation and its connection to tauopathy the design of functional nanomachines for drug delivery, molecular mechanisms of apoptosis, and structural characterization of an animal protein involved in lipid-transport.

Lyu et al. compared the structural properties of Tau35 (Wray et al., 2008), a truncated form of tau found in human brain in a subset of tauopathies (Guo et al., 2017), with the two longer isoforms 2N3R and 2N4R tau. Using small angle X-ray scattering, they found that Tau35 is more rigid than the other two isoforms. Additionally, Tau35 aggregates more quickly and to a greater extent than full-length tau in the presence of the polyanion heparin, an agent often used in tau studies to induce protein aggregation (Zhang et al., 2019; Lin et al., 2020). Combining several biophysical techniques they show that Tau35 aggregation is similar to previously reported tau fibrils but more densely packed. Their findings provide insight into the aggregation-inducing properties of clinically relevant tau fragments and their role in the pathogenesis of human tauopathies.

Protein assemblies can also be used to produce functional nanosystems for drug delivery. Ferritins (Fts) are ubiquitous proteins in nature and very versatile systems for biotechnology applications. The human heavy chain ferritin (hHFt) nanocages are ideal for the delivery of anti-cancer drugs due to their lack of immunogenicity and selective interaction with tumor cells (Li et al., 2010; Zhen et al., 2013). However, the current protocols for disassembling and reassembling hHFt to load cargo molecules have still some drawbacks, such as low protein recovery (Zhang et al., 2020; Zhang et al., 2021) and homogeneity. Moreover, the exposure to extreme pH during loading may damage the cargo molecule. Lucignano et al. present an alternative, efficient and reproducible procedure to reversibly disassemble hHFt under mild pH conditions using a small amount of sodium dodecyl sulphate (SDS). Electron microscopy and X-ray crystallography show that the reassembled protein is identical to the untreated one. This new procedure is much more versatile than existing protocols as it can be applied in a wide range of pH values and temperatures. Its versatility was demonstrated by encapsulation of two small molecules. Moreover, it is compatible with a larger number of cargos and allows for higher protein recovery compared to existing protocols.

Protein interactions are also central to apoptosis. This cellular process is the Research Topic of the review of Sora et al. that gives an accurate overview of structural details of the BH3 motif, which is found in many proteins involved in apoptosis. BH3 motifs are short motifs, not highly conserved in terms of sequence, but they typically contain two residues—a leucine and an aspartate—with a variable number of non-conserved residues in between (Aouacheria et al., 2015). The boundaries of BH3 motifs are not well-defined, with some of them spanning up to 20 residues (DeBartolo et al., 2014). Experimental and computational structural studies have shown that this motif has high structural plasticity. When it is isolated, it is mainly disordered in solution but readily adopts a mainly alpha helical fold when it binds partner proteins like BCL2 proteins. A thorough comprehension of the binding between BH3 motifs and their partner proteins is essential for both basic research and its practical applications. Disruption of the complexes between BCL2 proteins through competitive binding of small mimetics is the basis of various pharmaceutical drugs, some of which are already in clinical trials (Diepstraten et al., 2021).

Another type of transport has been shown for honeybee Vitellogenin (Vg), the most ancient yolk precursor protein in animals (Smolenaars et al., 2007). Vg is involved in lipid and other nutrients transport through developing embryos (Amdam et al., 2006; Salmela et al., 2015; Sun and Zhang, 2015). Leipart and coworkers present in this Research Topic a hypothesis about the function of the honeybee Vg based on the structural model of the protein derived from Alphafold and EM contour mapping. Vg is a glycolipophosphoprotein belonging to the large lipid transfer protein (LLTP) family. The full-length protein structure revealed a large hydrophobic lipid binding site and a well-defined fold at the C-terminal region. Leipart et al. propose a shielding mechanism that allows the C-terminal region of Vg to cover this large hydrophobic area exposed in the all-atom model. This mechanism is thought to influence lipid molecules’ uptake, transport, and delivery, and requires elasticity in the Vg lipid core, as described for homologous proteins in the large lipid transfer protein (LLTP) superfamily to which Vg belongs.

The publications in this Research Topic demonstrate that women in science should be a normality. This will only be achieved through encouraging gender equality, challenging of stereotypes, and motivating girls and women to pursue STEM careers.
